# Voreloxin Is an Anticancer Quinolone Derivative that Intercalates DNA and Poisons Topoisomerase II

**DOI:** 10.1371/journal.pone.0010186

**Published:** 2010-04-15

**Authors:** Rachael E. Hawtin, David E. Stockett, Jo Ann W. Byl, Robert S. McDowell, Nguyen Tan, Michelle R. Arkin, Andrew Conroy, Wenjin Yang, Neil Osheroff, Judith A. Fox

**Affiliations:** 1 Sunesis Pharmaceuticals, Inc., South San Francisco, California, United States of America; 2 Departments of Biochemistry and Medicine, Vanderbilt University School of Medicine, Nashville, Tennessee, United States of America; 3 3-V Biosciences, Palo Alto, California, United States of America; 4 Genentech, Inc., South San Francisco, California, United States of America; 5 Small Molecule Discovery Center, University of California San Francisco, San Francisco, California, United States of America; 6 Eiger Biopharmaceuticals, Inc., Palo Alto, California, United States of America; Mizoram University, India

## Abstract

**Background:**

Topoisomerase II is critical for DNA replication, transcription and chromosome segregation and is a well validated target of anti-neoplastic drugs including the anthracyclines and epipodophyllotoxins. However, these drugs are limited by common tumor resistance mechanisms and side-effect profiles. Novel topoisomerase II-targeting agents may benefit patients who prove resistant to currently available topoisomerase II-targeting drugs or encounter unacceptable toxicities. Voreloxin is an anticancer quinolone derivative, a chemical scaffold not used previously for cancer treatment. Voreloxin is completing Phase 2 clinical trials in acute myeloid leukemia and platinum-resistant ovarian cancer. This study defined voreloxin's anticancer mechanism of action as a critical component of rational clinical development informed by translational research.

**Methods/Principal Findings:**

Biochemical and cell-based studies established that voreloxin intercalates DNA and poisons topoisomerase II, causing DNA double-strand breaks, G2 arrest, and apoptosis. Voreloxin is differentiated both structurally and mechanistically from other topoisomerase II poisons currently in use as chemotherapeutics. In cell-based studies, voreloxin poisoned topoisomerase II and caused dose-dependent, site-selective DNA fragmentation analogous to that of quinolone antibacterials in prokaryotes; in contrast etoposide, the nonintercalating epipodophyllotoxin topoisomerase II poison, caused extensive DNA fragmentation. Etoposide's activity was highly dependent on topoisomerase II while voreloxin and the intercalating anthracycline topoisomerase II poison, doxorubicin, had comparable dependence on this enzyme for inducing G2 arrest. Mechanistic interrogation with voreloxin analogs revealed that intercalation is required for voreloxin's activity; a nonintercalating analog did not inhibit proliferation or induce G2 arrest, while an analog with enhanced intercalation was 9.5-fold more potent.

**Conclusions/Significance:**

As a first-in-class anticancer quinolone derivative, voreloxin is a toposiomerase II-targeting agent with a unique mechanistic signature. A detailed understanding of voreloxin's molecular mechanism, in combination with its evolving clinical profile, may advance our understanding of structure-activity relationships to develop safer and more effective topoisomerase II-targeted therapies for the treatment of cancer.

## Introduction

Type II topoisomerases are essential for the survival of eukaryotic cells [1], [2], [3], [4], [5]. These enzymes maintain DNA topology, disentangling DNA that becomes knotted, under- or over-wound in the process of replication, and are required to maintain correct chromosome condensation, decondensation, and segregation. Topoisomerase II acts by passing an intact DNA double helix through another double helix that has been cleaved by the enzyme, requiring a complex conformational change in the enzyme that is fueled by ATP hydrolysis [1], [3], [4], [6]. Following DNA strand passage, topoisomerase II religates the cleaved strand. Vertebrate cells encode two isoforms of topoisomerase II, α and β, [1], [3], [4], [5] which perform functions encompassing replication, transcription and DNA repair (reviewed in [5]). Topoisomerase IIα has been studied most extensively. This isoform is associated with replication and is essential for chromosomal segregation. Consistent with these functions its expression peaks at G2/M phase of the cell cycle [1], [3], [5], [7], [8].

**Figure 1:**
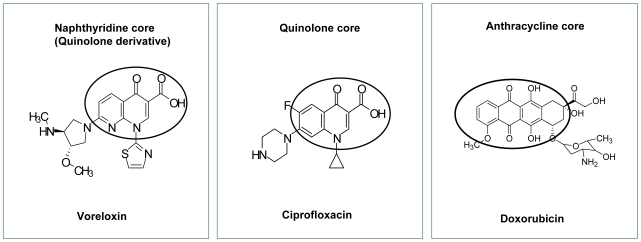
Voreloxin is a quinolone derivative. The chemical structures of voreloxin, ciprofloxacin and doxorubicin are shown. The core naphthyridine, quinolone, and anthracycline components are circled. Similarities between the core of the naphthyridine and quinolone are evident, as is the chemical dissimilarity between these two classes of compounds and the anthracyclines.

Topoisomerase II is well validated as a target of antineoplastic drugs that poison the enzyme [3], [9], [10], [11]. Poisons act by increasing the concentration of the covalent topoisomerase II-cleaved DNA reaction intermediate (i.e. cleavage complex), converting the transient DNA double-strand breaks (DSB) into permanent lesions, with catastrophic impact in replicating cells [3], [10]. Topoisomerase II poisoning may result by direct interaction of the drug with the enzyme, or by alterations in DNA structure [3], [9], [10], [11]. The widely used epipodophyllotoxins, etoposide and teniposide, do not intercalate DNA, but poison topoisomerase II by inhibiting religation [3], [9], [10]. Intercalative topoisomerase II-poisoning drugs include the anthracyclines doxorubicin (Figure 1), daunorubicin and idarubicin, and the anthracenedione, mitoxantrone. The anthracyclines and mitoxantrone are broadly used in the treatment of both solid and hematologic malignancies [3], [9], [10], but are limited in part by their sensitivity to P-glycoprotein (P-gp) receptor-mediated efflux [12], [13], [14].

In addition to intercalation and topoisomerase II poisoning, the anthracyclines interact with DNA in multiple ways, mediating DNA damage through non topoisomerase II-mediated mechanisms [15]–[16]. Principal scaffold-related cytotoxic activities of these drugs arise from induction of reactive oxygen species (ROS) that generate mutagenic base modifications with minimal site selectivity [17], [18]. ROS also stimulate cellular formaldehyde production, which in turn drives the formation of anthracycline DNA adducts and crosslinks [16], [19], [20], [21], [22], [23]. The relative roles of each of these processes in the clinical activity and toxicity of the compounds remains in debate [11], [15], [16]. The generation of ROS has been associated with the induction of cardiomyopathy which limits the lifetime cumulative anthracycline dose [15], [24], [25], [26]. In addition, oxidative DNA base damage that induces potentially mutagenic lesions was observed in blood samples from doxorubicin-treated patients [18]. These limitations, combined with susceptibility to P-gp-mediated drug resistance, have prompted the search both for third-generation anthracyclines and for alternatives to the anthracycline scaffold [11], to avoid such liabilities while retaining the efficacy of these broadly used drugs [16], [27], [28].

Quinolone-based drugs induce DNA damage in bacteria by poisoning bacterial DNA gyrase and topoisomerase IV, enzymes that are functional analogs of eukaryotic topoisomerase II [29], [30], [31], [32]. This led Tomita and coworkers to screen a number of antibacterial agents with quinolone-type ring structures for possible antineoplastic activity [33]. A class of compounds bearing a 1,8-naphthyridine core was subsequently optimized for cytotoxicity [34], resulting in the discovery of voreloxin (AG-7352), a novel naphthyridine analog (Figure 1). Voreloxin has no antibacterial activity, but exhibits potent cytotoxicity towards eukaryotic cancer cell lines [35], synergistic activity with cytarabine in acute myeloid leukemia (AML) cancer cell lines and supra-additivity in combination with cytarabine in a mouse model of bone marrow ablation and recovery [36]. Voreloxin activity was not affected by common mechanisms of drug resistance, including P-gp overexpression, when evaluated in etoposide- and anthracycline-resistant nonclinical models. These data, including cell-based and in vivo activity in 3 drug resistant cell lines, are previously published by Hoch et al [35]. In addition, objective responses were observed in patients for whom prior treatment with anthracyclines has failed [37], [38].

Here we establish the activity of voreloxin as a first-in-class topoisomerase II poison and inhibitor that intercalates DNA and induces site-selective DNA DSB, G2 arrest, and apoptosis. Using planar and nonplanar analogs, we determined that the intercalative properties of voreloxin are critical for its anticancer activities. In defining the voreloxin mechanism of action, these studies identify a novel chemical scaffold, distinct from the anthracyclines and epipodophyllotoxins, for development of topoisomerase II poisons that avoids resistance due to P-gp expression and possibly also the dose-limiting toxicities of the anthracyclines.

## Results

### Voreloxin is a topoisomerase II poison and induces site-selective DNA DSB mediated by human topoisomerase IIα and β

**Figure 2:**
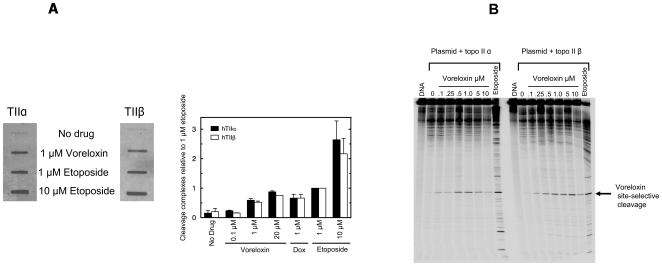
Voreloxin poisons topoisomerase II and induces site-selective DNA DSB. **A**, CCRF-CEM cells were untreated (No drug) or treated for 4 h with voreloxin (0.1–20 µM), doxorubicin (1 µM) or etoposide (1 or 10 µM), DNA harvested through a caesium chloride pad, DNA quantities normalized, and topoisomerase II levels analyzed following a slot blot using anti-topoisomerase IIα or β antibodies. The immunoblot is a representative of three independent experiments. Quantitative analysis was performed using the Alpha Innotech digital imaging system and each condition was compared relative to the level of cleavage complex induced by 1 µM etoposide. Error bars represent the standard deviations for three independent experiments. **B**, pBR322 was incubated in vitro with either purified topoisomerase IIα or β and a dose-titration of voreloxin (0.1–10 µM) or etoposide (5 µM). DNA cleavage was assessed using SDS-PAGE, with untreated reaction mix (0) or DNA alone (DNA) as controls. Densitometry quantification of the indicated band, and the sequence surrounding the cleavage site, are shown in Figure S2.

The ability of voreloxin to poison human topoisomerase II was evaluated in CCRF-CEM acute lymphocytic leukemia cells using the ICE bioassay [39]. This assay evaluates the amount of topoisomerase II stably associated with DNA (i.e., identifies the generation of stable cleavage complexes) by means of DNA isolation followed by immunoblot for detection of associated enzyme. As shown in Figure 2A, no cleavage complexes were detected after exposure to 0.1 µM voreloxin. One µM voreloxin drove the stable association with DNA of both topoisomerase IIα and β, with only a slight increase in cleavage complex formation at 20 µM. Densitometry readings indicated that the levels of cleavage complex induced by 1 µM voreloxin were equivalent to approximately one-half of those induced by 1 µM etoposide, and were comparable with those induced by 1 µM doxorubicin.

The induction of DNA DSB by voreloxin was established by pulsed-field gel electrophoresis following treatment of CCRF-CEM cells with a dose-titration of voreloxin. Dose-dependent induction of DNA fragmentation was detectable at the lowest (0.3 µM) concentration employed (Figure S1). In comparison with 0.1 µM doxorubicin, 1 µM voreloxin induced approximately equivalent DNA DSB.

The quinolone antibacterials interact with DNA and bacterial DNA gyrase and topoisomerase IV to induce DSB at preferred sequences [40]. To determine whether voreloxin recapitulates such activity, plasmid DNA was incubated with human topoisomerase IIα or β in the presence of a dose-titration of voreloxin, and reaction products were analyzed by gel electrophoresis. As shown in Figure 2B, dose-dependent fragmentation of DNA was observed with the production of a specific DNA fragment at all doses, in contrast with the DNA laddering induced by 1 µM etoposide.

The voreloxin cleavage product was quantified by densitometry and found to peak at voreloxin concentrations of 0.5 µM (topoisomerase IIα) or 1 µM (topoisomerase IIβ) and decline at higher concentrations, suggesting inhibition of enzymatic activity by higher drug concentrations (Figure S2). This may result from catalytic inhibition or, possibly, by limiting access of topoisomerase II to DNA as the amount of intercalated drug increases. These possibilities are currently under investigation. Sequencing of the specific cleavage fragment identified the cleavage site as GC/GG (Figure S2). This is consistent with the preferred cleavage site of quinolones, which induce DNA cleavage at and around G/C rich sequences [40] and in contrast with the topoisomerase II-mediated DNA DSB induced by doxorubicin, which favors a 3′A at the site of cleavage [41].

### Voreloxin-induced G2 arrest is partially dependent on topoisomerase II

**Figure 3:**
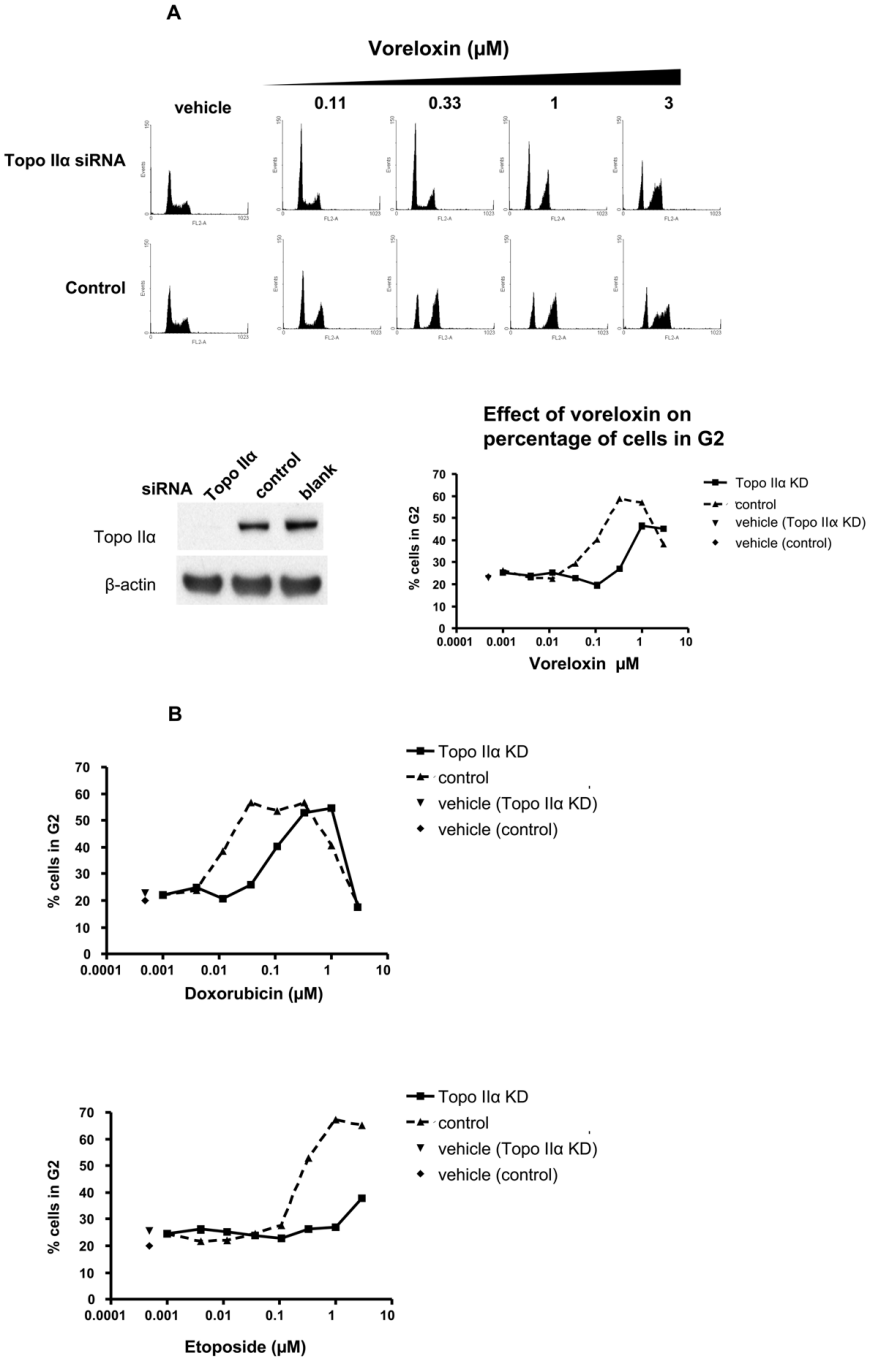
Topoisomerase II knockdown has a greater effect on G2 arrest induced by etoposide than by voreloxin or doxorubicin. **A**, A549 cells were transfected with siRNA targeting topoisomerase IIα (Topo IIα KD), or with scrambled control siRNA for 48 h, when a sample was taken to confirm the knockdown by Western blot. Blank  =  no siRNA. At 48 h, cells were treated for 16 h with a dose-titration (0.001–3 µM) of voreloxin and stained with BrdU followed by flow cytometry analysis. The percentages of cells in the G2 phase of the cell cycle, calculated from these data, are represented by the line graphs. Data are representative of 6 independent experiments. **B**, Cells were transfected and treated as described in (A) with a dose-titration (0.001–3 µM) of voreloxin, doxorubicin or etoposide. The same total transfected cell population was split and seeded for treatment with each drug. The percentages of cells in the G2 phase of the cell cycle are represented by the line graphs. Histograms are shown in Figure S3a, S3b, S3c. Data were consistent in 2 independent experiments.

The dependence of voreloxin on topoisomerase II for G2 arrest, a hallmark of topoisomerase II inhibition [42], was investigated in the A549 lung cancer cell line using siRNA knockdown of topoisomerase IIα (the isoform associated with replication and essential for chromosome segregation). The level of knockdown shown in Figure 3A is representative of 6 experiments performed using this approach. The extent of topoisomerase IIα knockdown was maximal at 48 h, at which time the cells were treated with titrated amounts of voreloxin for 16 h. The relatively short duration of this assay allowed analysis to be completed within the timeframe of a transient topoisomerase IIα knockdown. Cells with reduced topoisomerase IIα showed reduced sensitivity to voreloxin-induced G2 arrest. Maximal arrest was observed at 1 µM in the cells with reduced levels of topoisomerase IIα (47% of cells in G2) whereas 0.11 µM voreloxin in the control cells achieved comparable arrest (40% of cells in G2) and was maximal at 0.33 µM (59% of cells in G2) (Figure 3A, histograms of complete dose-range are shown in Figure S3a and a summary of additional experiments is shown in Table S1).

The effect of reduced topoisomerase IIα expression on voreloxin-induced G2 arrest was compared with the effect on doxorubicin- and etoposide-induced G2 arrests in the same transfected cell population (Figure 3B, raw histograms in Figure S3b and Figure S3c, respectively, and a summary of data from additional experiments is shown in Table S2). When topoisomerase IIα levels were reduced, the induction of G2 arrest in doxorubicin-treated cells shifted from a maximum at 0.037 µM in control cells (57% of cells in G2) to 0.33 µM (53% of cells in G2). Thus, the magnitude of desensitization to drug-induced G2 arrest by topoisomerase IIα knockdown was comparable for voreloxin and doxorubicin. Etoposide activity was more dependent upon topoisomerase IIα expression (Figure 3B). The G2 arrest (53% of cells in G2) was evident at 0.33 µM in control cells whereas a lower level of arrest (38% of cells in G2) was evident only at 3 µM etoposide in topoisomerase IIα knockdown cells.

### Voreloxin does not generate significant levels of ROS

Because ROS contribute to the DNA damage induced by the anthracyclines, the production of ROS by voreloxin was investigated and compared with that of doxorubicin. HCT-116 colon cancer cells were treated for 6 h with a dose-titration of either voreloxin (1–9 µM) or doxorubicin (0.03–2 µM) in the presence of a ROS indicator (2′,7′- dichlorofluorescein which fluoresces when oxidized). As shown in Figure S4, voreloxin did not produce significant levels of ROS in comparison with doxorubicin. These observations are consistent with voreloxin's less chemically reactive quinolone-based structure [29].

### Voreloxin cytotoxic activity requires DNA intercalation

**Figure 4:**
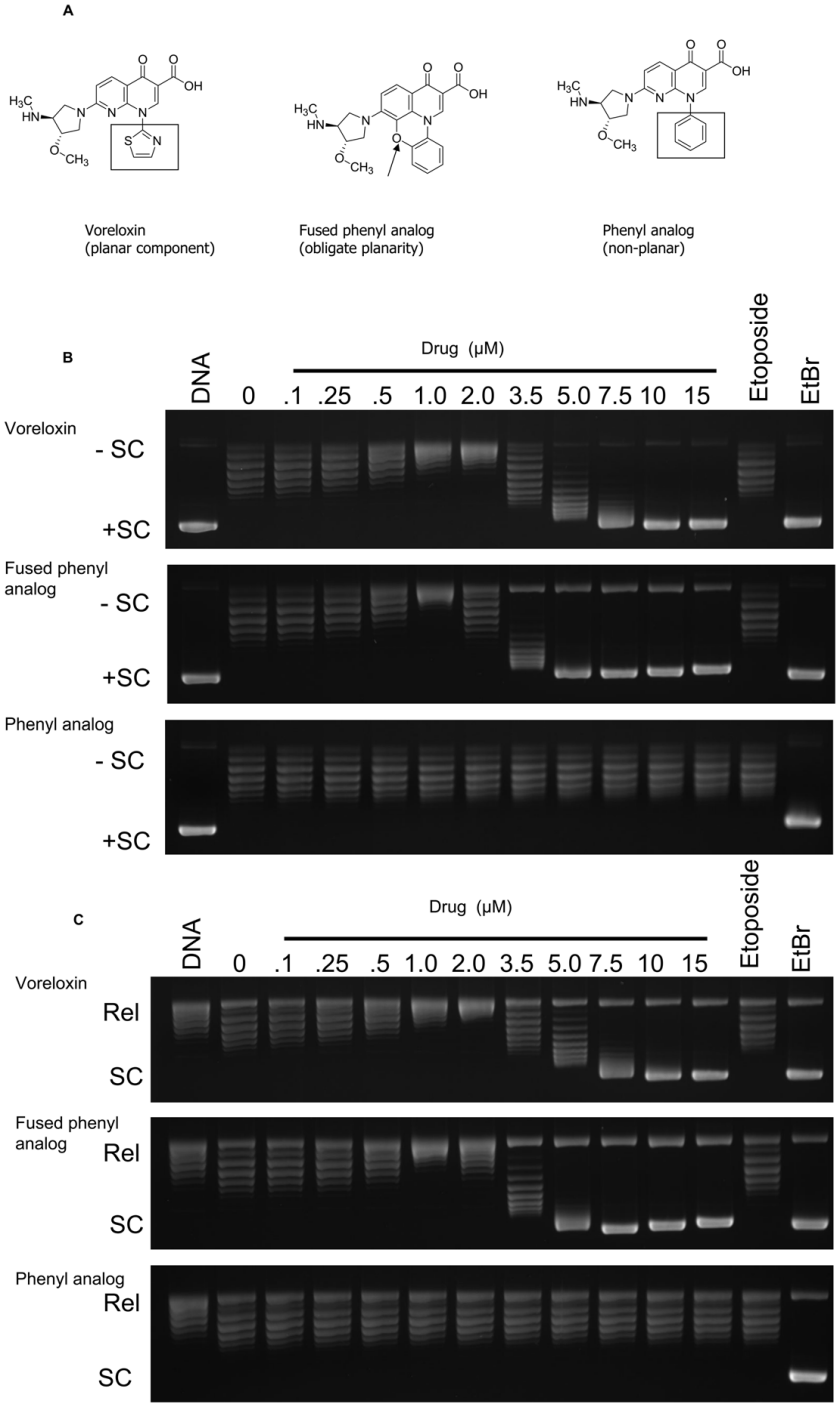
Voreloxin and the fused phenyl analog intercalate DNA, while the nonplanar phenyl analog does not. **A**, Structures of voreloxin and the two analogs are shown, with the thiazole group of voreloxin boxed and the fused phenyl and phenyl rings highlighted by arrow or box, respectively. **B and C**, Agarose gels stained with ethidium bromide are shown. Intercalation was evaluated by conversion of negatively supercoiled DNA (-SC) into positively supercoiled DNA (+SC) (**4B**) or the conversion of relaxed plasmid DNA (Rel) to supercoiled molecules (SC) (**4C**). Control reactions were carried out in the absence of both drug and enzyme (labeled as DNA) or in the absence of drug but containing enzyme (labeled 0). Topoisomerase I concentration was constant. Reactions containing etoposide (100 µM) and ethidium bromide (10 µM) are included as examples of a nonintercalative and intercalative drug, respectively. Data were consistent in two independent experiments.

Structure-activity studies of 1,8-naphthyridine analogs suggested that coplanarity of the naphthyridine core and the N-1 thiazole ring was required for antineoplastic activity. In earlier studies of voreloxin analogs, replacing the thiazole ring with a phenyl ring led to a 100-fold reduction in activity [33]. Based on electronic structure analysis, we attributed this loss in activity to the need for the phenyl ring to twist out-of-plane to avoid steric conflicts. The relationship of molecular planarity to intercalation potential was probed using two structural analogs of voreloxin (Figure 4A). The N-1 phenyl compound was synthesized as a nonplanar comparator, while a fused analog was generated to enforce planarity of the aromatic system. The intercalative properties of the three compounds were evaluated in a topoisomerase I intercalation assay, using either negatively supercoiled or relaxed DNA as the substrate. As shown in Figure 4B and 4C, voreloxin intercalation of DNA was detectable at 1 µM, and at 10 µM full intercalation was observed. No intercalation of the phenyl derivative was identified, whereas the fused phenyl analog intercalated DNA to a greater extent than voreloxin, and was maximal by 5 µM.

**Figure 5:**
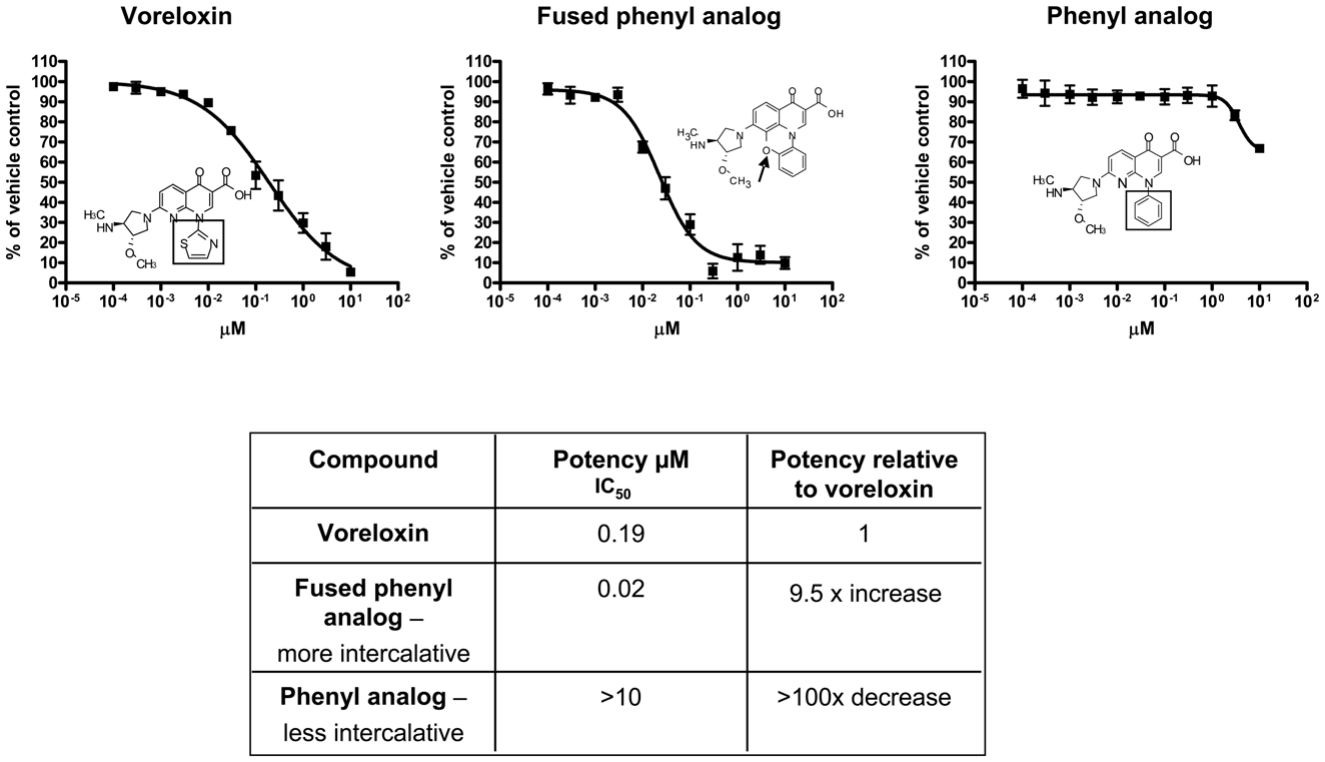
Cytotoxicity correlates with the ability of voreloxin and analogs to intercalate DNA. A549 cells were treated for 72 h with a dose-titration (0.0001–10 µM) of voreloxin or analog, each treatment point performed in triplicate, N = 2, and the inhibition of proliferation analyzed by MTT assay. Error bars represent standard error of the mean (SEM) for the two independent experiments. The potency of the analogs relative to voreloxin was compared using IC_50_ values. The compound structures are inset, with the fused phenyl and phenyl rings highlighted by arrow or box, respectively.

To correlate cytotoxicity with the intercalative potential of voreloxin and the structural analogs, their activities were compared in both proliferation (Figure 5) and colony forming assays (Figure S5). No IC_50_ could be established for the nonintercalative phenyl derivative, due to weak and absent cytotoxicity in the proliferation and colony growth inhibition assays, respectively (Figure 5 and Figure S5). In contrast, the intercalative fused phenyl analog was consistently more cytotoxic than voreloxin in both proliferation and colony growth inhibition assays (Figure 5 and Figure S5). A comparison of the inhibition of proliferation by the two compounds showed an average 9.5-fold increase in potency over voreloxin for the planar fused phenyl intercalative analog (Figure 5). These data are representative of proliferation inhibition data obtained in three additional human cancer cell lines: HCT-116 and HT-29 (colon cancer) and K562 (chronic myelocytic leukemia).

**Figure 6:**
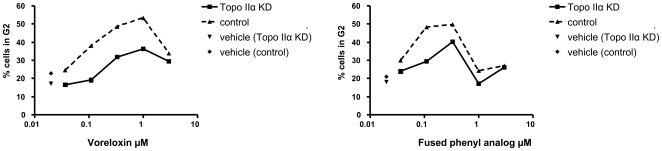
Topoisomerase IIα knockdown reduces the G2 arrest induced by the planar voreloxin analog. A549 cells were transfected with siRNA targeting topoisomerase IIα (Topo IIα KD), or with scrambled control siRNA, for 48 h. At 48 h, cells were treated for 16 h with a dose-titration (0.037–3 µM) of voreloxin or analog and stained with BrdU followed by flow cytometry analysis. Histograms are shown in Figure S6. The percentages of cells in the G2 phase of the cell cycle were calculated and are represented in the line graphs. No G2 arrest was observed with the nonintercalative analog (Figure S6 and Table S3). Data are representative of three independent experiments.

No G2 arrest was observed with the phenyl nonintercalative analog (Figure S6 and Table S3). Dose-dependent induction of G2 arrest by the planar fused phenyl intercalative analog was established, and the effect of topoisomerase IIα knockdown on the induction of G2 arrest was compared with voreloxin in the same population of siRNA transfected cells. Desensitization to voreloxin consistent with previous data, as well as with the planar fused phenyl analog, was observed in cells with reduced levels of topoisomerase IIα (Figure 6, raw histograms in Figure S6). The planar fused phenyl analog induced G2 arrest in control cells at 0.11 µM (48% of cells in G2) and at 0.33 µM (40% of cells in G2) in topoisomerase IIα knockdown cells. Consistent with the enhanced cytotoxicity of this analog, a greater percentage of sub-G1 cells was identified at ≥1 µM, regardless of topoisomerase IIα knockdown (Figure S6). Repeats of this analysis are shown in Table S3. These data suggest that the cytotoxicity of the fused phenyl analog is less dependent than voreloxin upon topoisomerase II expression, and that enhanced intercalation has a greater impact on DNA structure and processing.

## Discussion

Voreloxin is a first-in-class quinolone derivative currently completing Phase 2 clinical trials in AML and platinum-resistant ovarian cancer. Here we establish that voreloxin intercalates DNA and poisons topoisomerase II, inducing site-selective DNA DSB and G2 arrest. The structures of voreloxin and the prototype fluoroquinolone antibacterial, ciprofloxacin, are illustrated in Figure 1. We used this structural similarity to help frame further mechanistic studies of voreloxin, and to guide comparison with other classes of antineoplastic agents in clinical use that cause DNA damage by interfering with topoisomerase II function.

The peak in voreloxin-induced DNA fragmentation at 1 µM, declining at higher concentrations, is consistent with the bell-shaped curve of DNA cleavage seen with intercalative topoisomerase II poisons [43], [44] and demonstrates that, although voreloxin retains many of the structural features of the quinolones, it has enhanced ability to intercalate double-stranded DNA [30], [45]. The peak in formation of cleavage complexes at approximately 1 µM voreloxin is in keeping with the observed concentration range for saturation in the DNA fragmentation and intercalation assays and represents the plasma concentration maintained for over 24 hours in treated patients [38]. The concomitant loss of cytotoxicity and intercalation in the phenyl voreloxin analog, and the increased cytotoxicity of the more intercalative fused phenyl analog, suggests the requirement for intercalation for anticancer cell cytotoxicity of quinolone analogs. In addition, topoisomerase IIα knockdown had a greater impact on the ability of etoposide (nonintercalative) to induce G2 arrest than on the G2 arrest activities of voreloxin, the planar fused phenyl analog, or doxorubicin. The impact of enhanced intercalation on molecular mechanism, which manifested in more potent cytotoxicity and reduced topoisomerase II dependence, is under investigation.

Because of similarities in mechanism of action, specifically both DNA intercalation and topoisomerase II poisoning, the anthracyclines are effective to help guide indication selection for voreloxin [15], [16]. In combination with cytarabine (the “7+3” treatment schedule), the anthracyclines are the standard of care in the treatment of newly diagnosed AML, and are broadly used in the treatment of other hematologic malignancies, as well as for breast and ovarian cancers [25]. Despite the efficacy of anthracycline-based therapies, structure-based toxicities limit their use, particularly given the cardiomyopathy that is associated with cumulative dose [15], [16], [25], [26]. The generation of ROS has been linked to the cardiotoxicity of the drugs [15] and also has been shown to induce potentially mutagenic DNA base lesions [18] in addition to driving the generation of DNA adducts and crosslinks [16], [19], [20]. In contrast, the quinolone core of voreloxin is less chemically reactive. As reported here, voreloxin does not generate significant ROS in cell-based studies, and the formation of ROS or DNA alkylation are not associated with the activity of the core quinolone structure. Dose-limiting toxicities of voreloxin are reversible oral mucositis (AML) [46], [47] and neutropenia (solid tumors) [37]. The contrasting structures of voreloxin and the anthracycline family member, doxorubicin, are shown in Figure 1.

The ability to induce site-selective DNA damage at GC/GG regions distinguishes voreloxin from both the anthracyclines and etoposide. By analogy with the quinolones, this may reflect a consequence of sequence-selective DNA cleavage by topoisomerase II in the context of the drug/DNA/enzyme complex [40], [45]. These targeted DNA-enzyme interactions contrast with the mechanistically less targeted [11], [15], [16], [18], [19], [20], [21], [22], [23] and highly intercalative [48] anthracyclines in current clinical use. The ability of voreloxin to intercalate double-stranded DNA in the absence of topoisomerase II suggests that the intercalative properties of the molecule are greater than those of the quinolone antibacterials [29], [30], [45].

Efficacy of both the anthracyclines and etoposide is hampered by sensitivity to the common tumor resistance mechanism of P-gp efflux [12], [13], [14]. In contrast, voreloxin is not a P-gp substrate [35] and has potent activity in nonclinical models of anthracycline- and etoposide-resistance that include overexpression of P-gp [35]. In addition, voreloxin was active against primary tumor biopsies resistant to doxorubicin or etoposide or both, and objective responses were observed in patients with relapsed/refractory AML and platinum-resistant ovarian cancer for whom anthracycline-based therapies have failed [37], [38].

The identification of the quinolone scaffold as a source for novel topoisomerase II poisons, combined with recent progress in our understanding of topoisomerase II biology, structure and function [5], [11], [49], provides a rationale to further examine this new family of anticancer therapeutics. For example, exploring topoisomerase II isozyme selectivity, investigating the sensitivity and resistance of topoisomerase II mutants to voreloxin and analogs, and possibly mapping the sites of drug–enzyme interaction may allow further optimization of topoisomerase II-targeting therapeutics to enhance clinical benefit.

In summary, these data establish voreloxin as a first-in-class quinolone analog that exerts potent anticancer activity through a mechanism that parallels the activity of the quinolones in bacterial cells, namely interaction with DNA and topoisomerase II poisoning. Voreloxin displays increased intercalation into double-stranded DNA compared to the antibacterials, and topoisomerase II poisoning that induces site-selective DNA DSB in GC rich regions. Based upon both chemical and mechanistic differences reported here, voreloxin may provide clinical advantages over other topoisomerase II poisons that are currently in use. Continued interrogation of the molecular and cellular activities of voreloxin and other members of this new family of quinolone derivatives as potential anticancer therapeutics is the focus of ongoing research.

## Materials and Methods

### Enzymes and materials

Recombinant wild-type human topoisomerase IIα and IIβ were expressed in *Saccharomyces cerevisiae* and purified as described previously[50]. Negatively supercoiled pBR322 DNA was prepared from *Escherichia coli* using a Plasmid Mega Kit (Qiagen) as described by the manufacturer.

### Formation of topoisomerase II-DNA cleavage complexes in cultured human cells

Human CEM leukemia cells were cultured under 5% CO_2_ at 37°C in RPMI 1640 medium (Cellgro by Mediatech, Inc.), containing 10% heat-inactivated bovine calf serum (Hyclone) and 2 mM glutamine (Cellgro by Mediatech, Inc.). The in vivo complex of enzyme (ICE) bioassay was modified as noted on the TopoGen, Inc. web site and detailed in Methods S1 [51], [52].

### Site-specific DNA cleavage mediated by topoisomerase II

DNA cleavage sites were mapped using a modification of the procedure of O'Reilly and Kreuzer [53] as detailed in Methods S1.

### DNA intercalation

Intercalation reaction mixtures contained 16.5 nM topoisomerase I and 5 nM relaxed or negatively supercoiled pBR322 DNA in a total of 20 µL of 50 mM Tris-HCl (pH 7.9), 0.1 mM EDTA, 50 mM KCl, 10 mM MgCl_2_, 0.5 mM DTT and 30 µg/mL BSA that contained 0–15 µM voreloxin or analogs. Mixtures were incubated at 37°C for 6 min, extracted with phenol:chloroform:isoamyl alcohol (25:24:1), and added to 3 µL of 0.77% SDS, 77 mM EDTA (pH 8.0). Samples were mixed with 2 µL of agarose gel loading buffer, heated at 45°C for 5 min, and subjected to electrophoresis in a 1% agarose gel in 100 mM Tris-borate (pH 8.3), 2 mM EDTA. Gels were stained with 1 µg/mL ethidium bromide, and DNA bands were visualized by ultraviolet light using an Alpha Innotech digital imaging system. Interpretation of these data are further explained in Methods S1.

### Cell cycle analysis

A549 cells were treated with 0.001–3 µM drug diluted in RPMI 1640 growth media containing 10% fetal calf serum for 16 h. Following treatment, adherent and floating cells were harvested, washed with PBS, fixed and stained for total DNA content based on propidium iodide fluorescence and cell cycle analysis performed by FACS as described in Methods S1.

### siRNA knockdown

A549 cells were transfected with 75 nM topoisomerase IIα-targeting siRNA (Dharmacon (Thermo Scientific) TOP2A ON-TARGETplus SMARTpool # L-004239-00-0005) combined with Lipofectamine 2000 (Invitrogen) in RPMI 1640 growth media containing 10% fetal calf serum, as recommended by the manufacturers. Additional control samples included cells transfected with nontargeting siRNA (Dharmacon (Thermo Scientific) ON-TARGETplus Non-targeting Pool # D-001810-10-05) and cells treated with Lipofectamine 2000 alone. After 24 h, cells were harvested with 0.1% trypsin-EDTA and seeded in 12-well dishes at 50,000 cells/well in normal growth media. Following an additional 24 h of growth (48 h following the initial exposure to siRNA), cells were treated with a dose-titration of voreloxin, doxorubicin, etoposide, or voreloxin analog.

### Western blot

Cells from each of the transfection conditions were harvested, lysed in M-PER buffer (Pierce), and topoisomerase IIα protein levels determined by Western blot analysis using a topoisomerase IIα specific antibody (Abcam) as described in Methods S1. Beta-actin was used as normalizing control.

### Measurement of cell proliferation by MTT

Cells were plated in 96-well plates at 1000 cells/well and treated with compound or vehicle control (0.1% final concentration) for 72 h using a 3-fold dose-titration. After treatment, MTT reagent (5 mg/mL, Sigma-Aldrich) was added directly to the media and incubated at 37°C/5% CO_2_ for 2 h. MTT lysis buffer was added and cells were incubated at 37°C/5% CO_2_ overnight. Samples were analyzed by measuring the light absorbance at 595 nm using the SpectraMax plate reader (Molecular Devices). Values obtained for treatment samples were normalized to control samples.

## Supporting Information

Figure S1 CCRF-CEM cells were treated for 6 h with a dose-titration of voreloxin (0.3 - 9 µM), 0.1 µM doxorubicin, or vehicle control, harvested and analyzed by PFGE. Fragmented DNA is detectable as indicated. MW  =  molecular weight marker. 0 =  untreated cells. Veh  =  vehicle.
(2.57 MB TIF)

Figure S2 Densitometry analysis and sequence determination of specific voreloxin cleavagae product (identified in Figure 2B). The sequencing image is a representative of three independent experiments. The quantitative analysis of the indicated voreloxin cleavage product is shown relative to untreated control using the Bio Rad Molecular Imager FX and the error bars represent three independent experiments. Sequencing of this product identified the site-selective cleavage sequence shown above the cleavage complex bar graph.
(0.42 MB TIF)

Figure S3 Histograms showing the effect of voreloxin, doxorubicin or etoposide on G2 arrest, in control cells or cells with reduced topoisomerase IIα. A549 cells were transfected with siRNA targeting topoisomerase IIα or with scrambled control siRNA for 48 h, when they were treated for 16 h with a dose-titration (0.001–3 µM) of (a) voreloxin, (b) doxorubicin, or (c) etoposide and stained with BrdU followed by flow cytometry analysis.
(2.73 MB TIF)

Figure S4 Comparison of ROS generation by voreloxin and doxorubicin. a. HCT116 cells were treated for 6 hours with a dose-titration of voreloxin (0.3–3 µM, upper panel), doxorubicin (0.1–1 µM, lower panel), or vehicle only negative control, in the presence of 2′,7′-dichlorofluorescein (DCF). ROS production was evaluated by FACS detection of oxidized fluorescent DCF reagent, comparing unstained cells (background fluorescence) with treated cells, counting 5000 events per treatment. b. Cells were treated as in (a) with voreloxin (1–9 µM), doxorubicin (2 µM), hydrogen peroxide positive control (H_2_0_2_ at 400 µM) or vehicle only negative control. ROS production was evaluated as in (a).
(8.05 MB TIF)

Figure S5 Colony growth inhibition induced by voreloxin and analogs. A549 cells were treated for 24 h with a dose-titration (0.03–3 µM) of voreloxin or analog, with each treatment point performed in triplicate. Cells were washed and seeded for analysis of colony growth inhibition as described. Data represent colonies detectable following 5 days growth, N = 2. Error bars represent SEM of the two independent experiments.
(0.88 MB TIF)

Figure S6 Histograms showing the effect of voreloxin or analogs on G2 arrest in control cells or cells with reduced topoisomerase IIα. A549 cells were transfected with siRNA targeting topoisomerase IIα or with scrambled control siRNA for 48 h, when they were treated for 16 h with a dose-titration (0.037–3 µM) of voreloxin or analogs.
(1.64 MB TIF)

Table S1 Summary of additional five experiments investigating the effect of topoisomerase IIα knockdown on voreloxin-induced G2 arrest. Indicated for each treatment group are the drug concentrations at which maximal G2 arrest was observed, and the percentage of cells in G2 at maximal arrest for each treatment group. Each experiment was performed independently.
(0.12 MB PPT)

Table S2 Summary of additional two experiments investigating the effect of topoisomerase IIα knockdown on doxorubicin and etoposide-induced G2 arrests. Indicated for each treatment group are the drug concentrations at which maximal G2 arrest was observed, and the percentage of cells in G2 at maximal arrest for each treatment group. Each experiment was performed independently.
(0.12 MB PPT)

Table S3 Summary of additional two experiments investigating the effect of topoisomerase IIα knockdown on the planar analog and phenyl analog-induced G2 arrests. Indicated for each treatment group are the drug concentrations at which maximal G2 arrest was observed, and the percentage of cells in G2 at maximal arrest for each treatment group. Each experiment was performed independently.
(0.12 MB PPT)

Methods S1 (0.04 MB DOC)
