# Supplementary figures and images for: Voreloxin Is an Anticancer Quinolone Derivative that Intercalates DNA and Poisons Topoisomerase II
Source: PLoS One. 2010 Apr 15;5(4):e10186. doi: 10.1371/journal.pone.0010186 (PMC2855444; doi:10.1371/journal.pone.0010186)

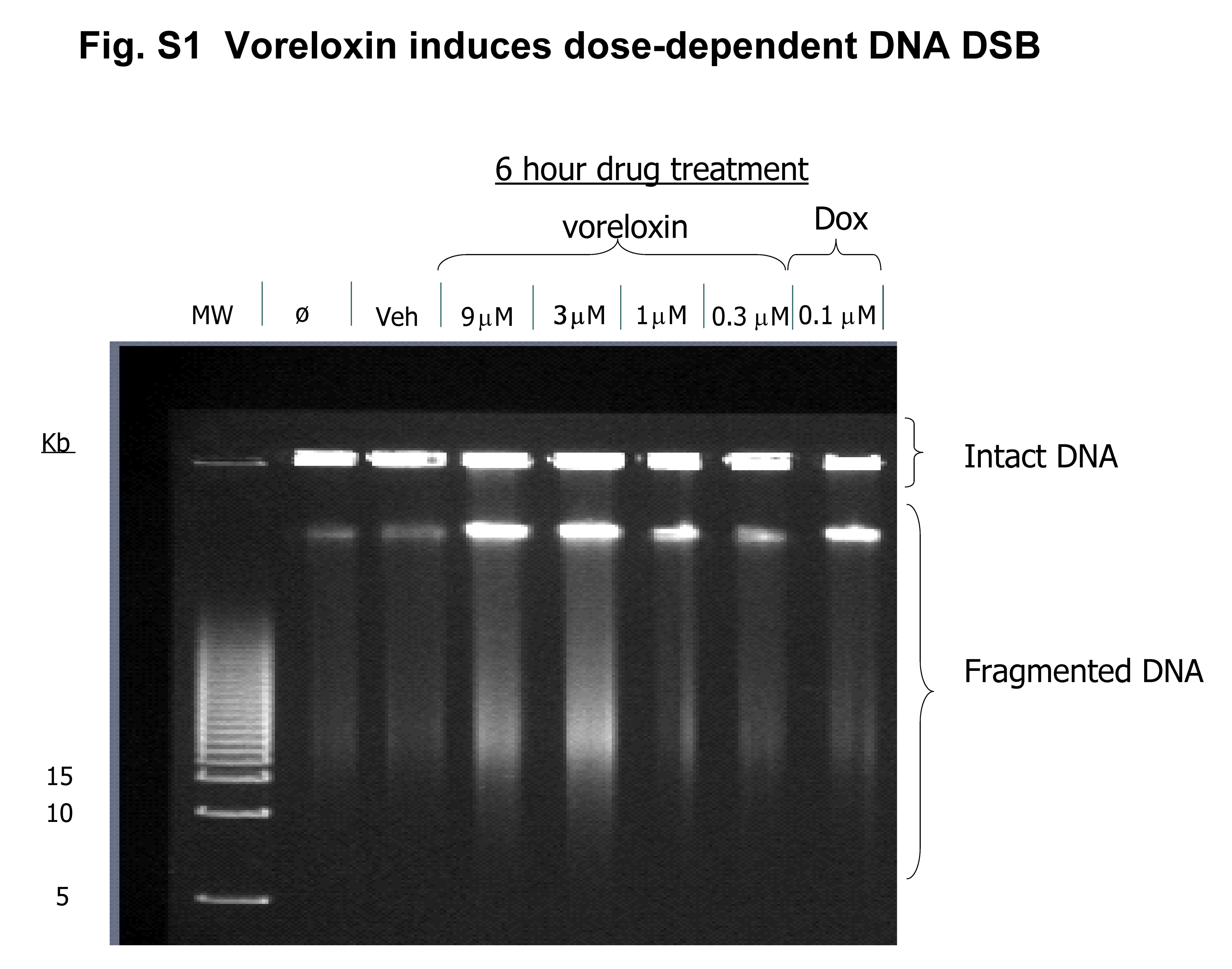

Supplement: Figure S1 — CCRF-CEM cells were treated for 6 h with a dose-titration of voreloxin (0.3 - 9 µM), 0.1 µM doxorubicin, or vehicle control, harvested and analyzed by PFGE. Fragmented DNA is detectable as indicated. MW = molecular weight marker. 0 = untreated cells. Veh = vehicle. (2.57 MB TIF) [file pone.0010186.s001.tif]

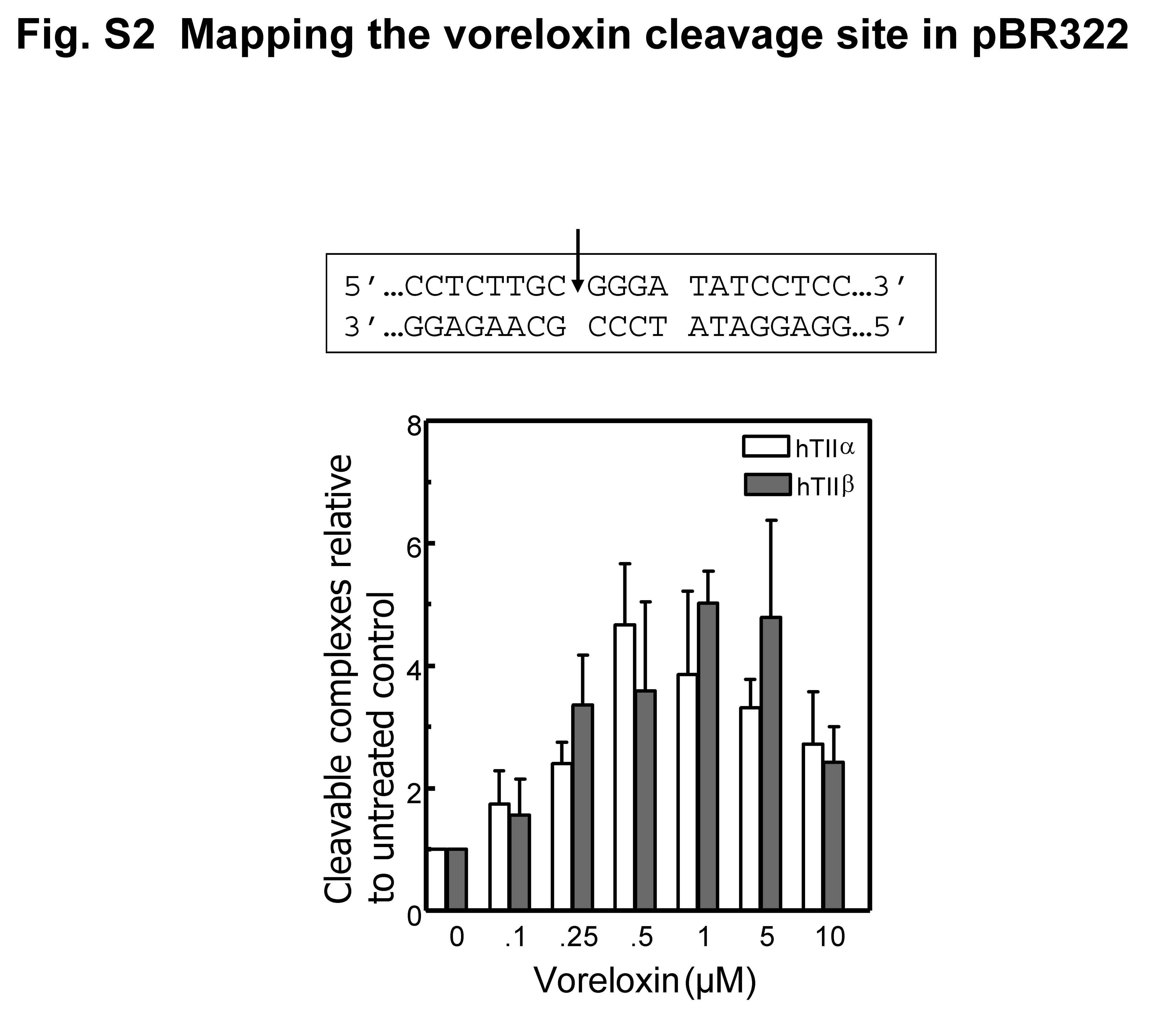

Supplement: Figure S2 — Densitometry analysis and sequence determination of specific voreloxin cleavagae product (identified in Figure 2B). The sequencing image is a representative of three independent experiments. The quantitative analysis of the indicated voreloxin cleavage product is shown relative to untreated control using the Bio Rad Molecular Imager FX and the error bars represent three independent experiments. Sequencing of this product identified the site-selective cleavage sequence shown above the cleavage complex bar graph. (0.42 MB TIF) [file pone.0010186.s002.tif]

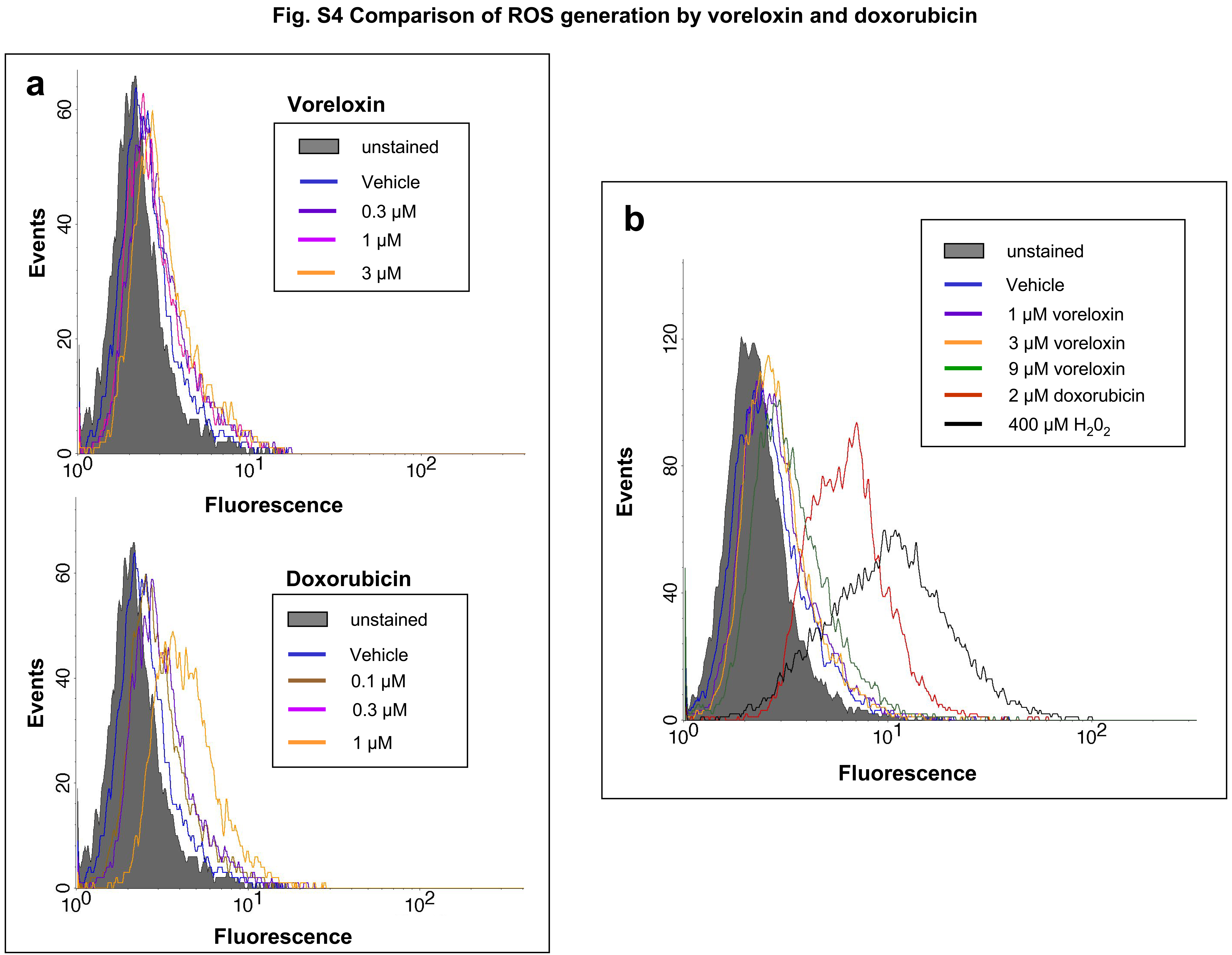

Supplement: Figure S4 — Comparison of ROS generation by voreloxin and doxorubicin. a. HCT116 cells were treated for 6 hours with a dose-titration of voreloxin (0.3–3 µM, upper panel), doxorubicin (0.1–1 µM, lower panel), or vehicle only negative control, in the presence of 2′,7′-dichlorofluorescein (DCF). ROS production was evaluated by FACS detection of oxidized fluorescent DCF reagent, comparing unstained cells (background fluorescence) with treated cells, counting 5000 events per treatment. b. Cells were treated as in (a) with voreloxin (1–9 µM), doxorubicin (2 µM), hydrogen peroxide positive control (H202 at 400 µM) or vehicle only negative control. ROS production was evaluated as in (a). (8.05 MB TIF) [file pone.0010186.s004.tif]

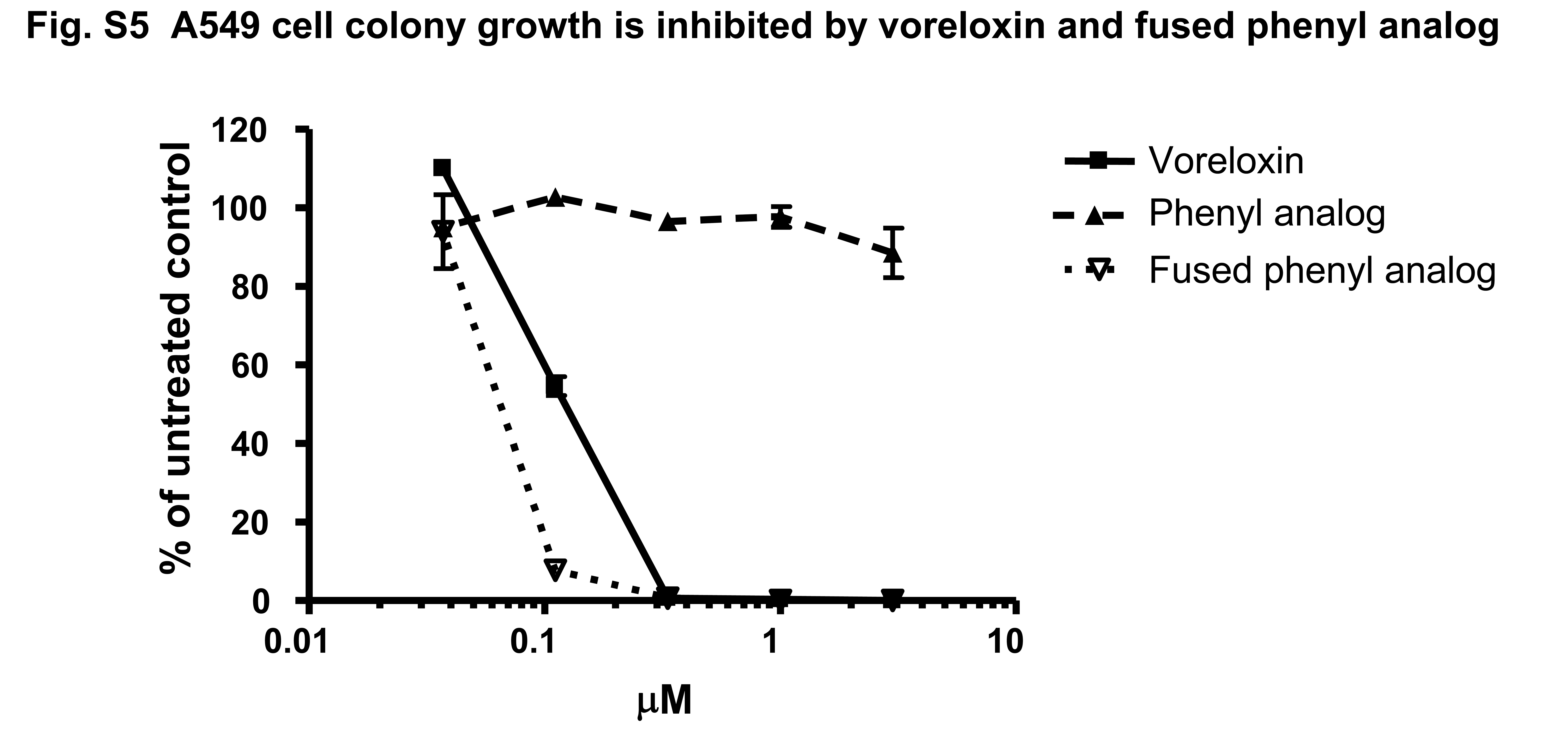

Supplement: Figure S5 — Colony growth inhibition induced by voreloxin and analogs. A549 cells were treated for 24 h with a dose-titration (0.03–3 µM) of voreloxin or analog, with each treatment point performed in triplicate. Cells were washed and seeded for analysis of colony growth inhibition as described. Data represent colonies detectable following 5 days growth, N = 2. Error bars represent SEM of the two independent experiments. (0.88 MB TIF) [file pone.0010186.s005.tif]

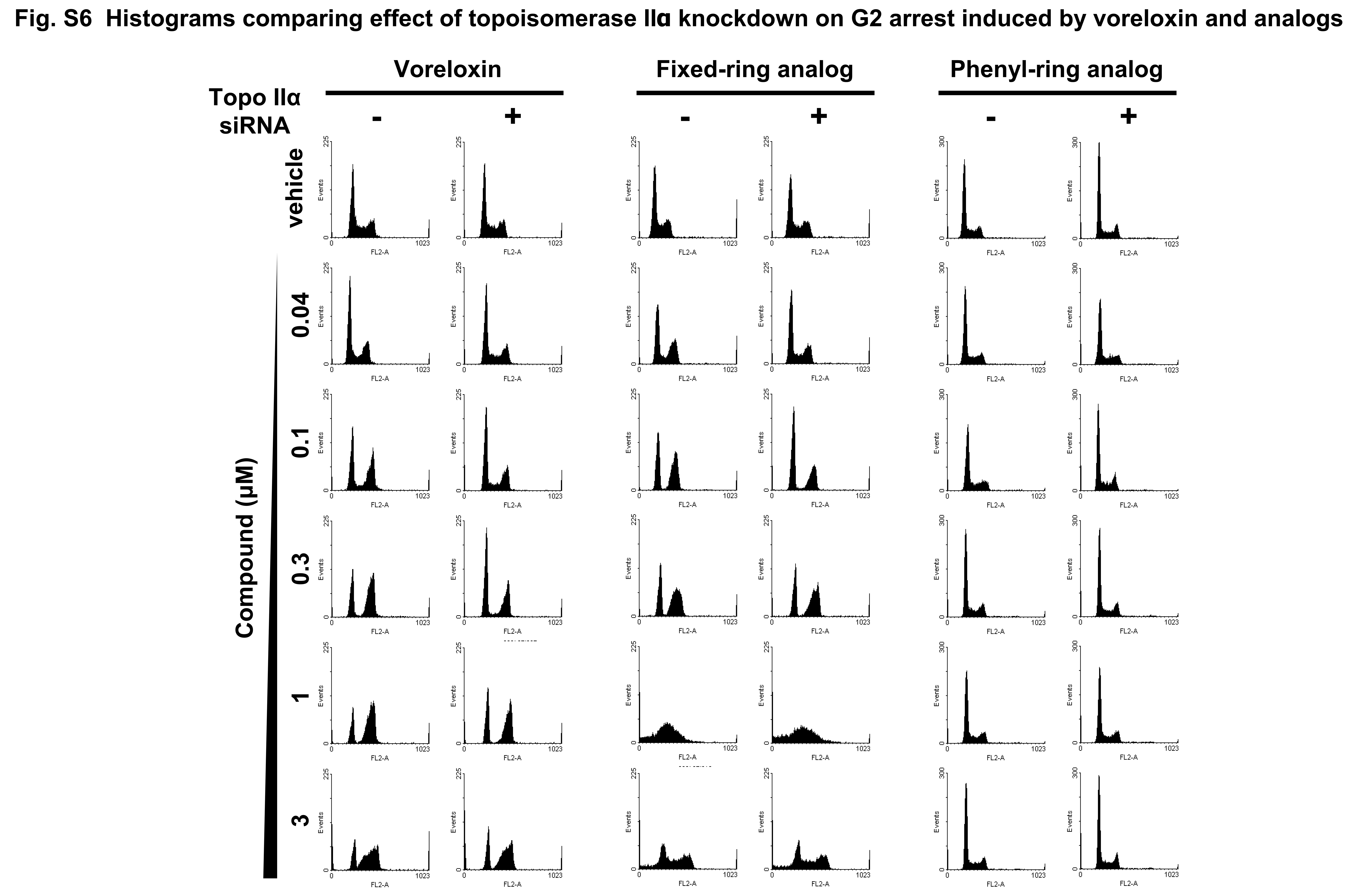

Supplement: Figure S6 — Histograms showing the effect of voreloxin or analogs on G2 arrest in control cells or cells with reduced topoisomerase IIα. A549 cells were transfected with siRNA targeting topoisomerase IIα or with scrambled control siRNA for 48 h, when they were treated for 16 h with a dose-titration (0.037–3 µM) of voreloxin or analogs. (1.64 MB TIF) [file pone.0010186.s006.tif]

## Slide 1
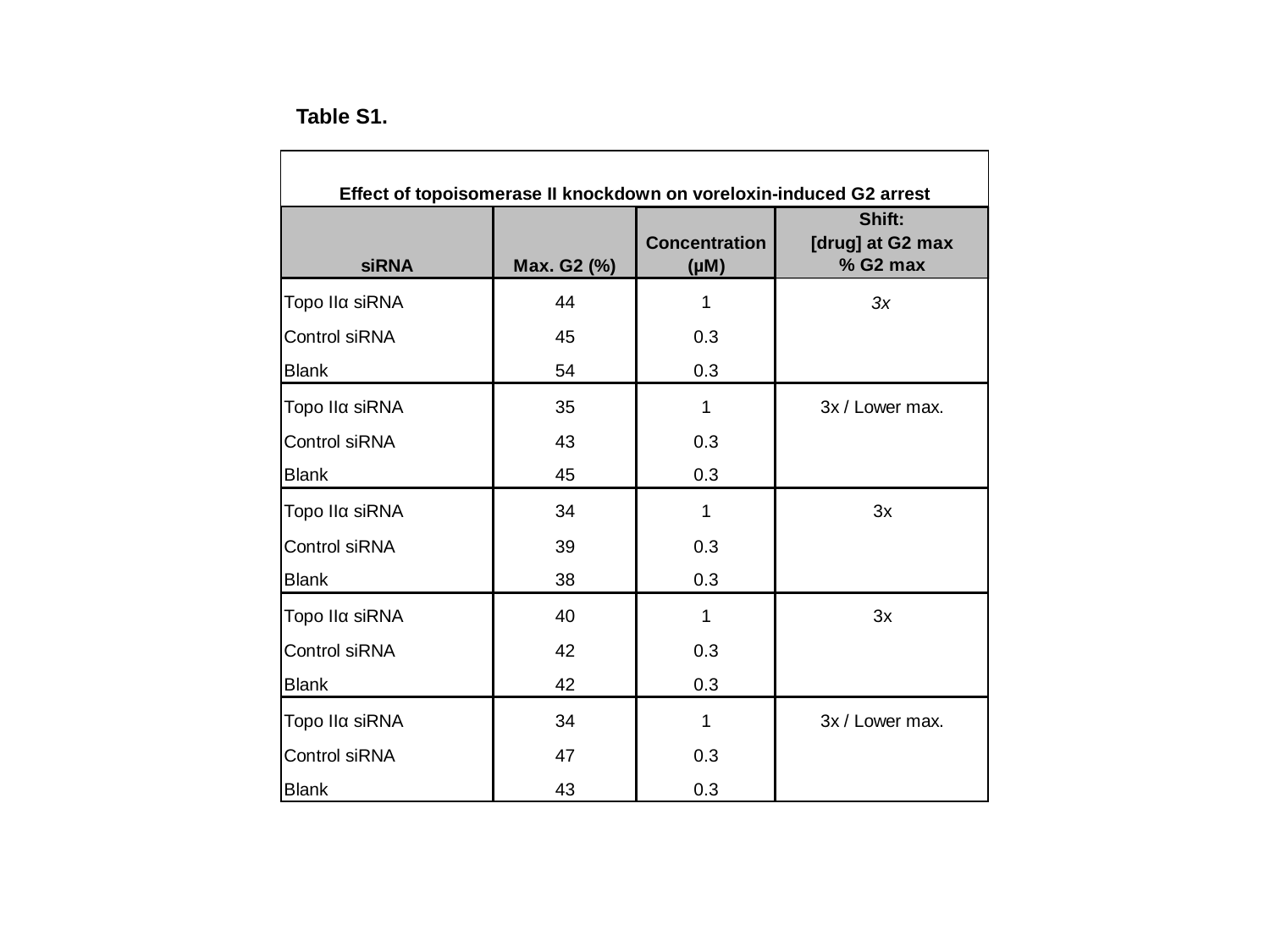

Table S1.

Supplement: Table S1 — Summary of additional five experiments investigating the effect of topoisomerase IIα knockdown on voreloxin-induced G2 arrest. Indicated for each treatment group are the drug concentrations at which maximal G2 arrest was observed, and the percentage of cells in G2 at maximal arrest for each treatment group. Each experiment was performed independently. (0.12 MB PPT) [file pone.0010186.s007.ppt]

## Slide 1
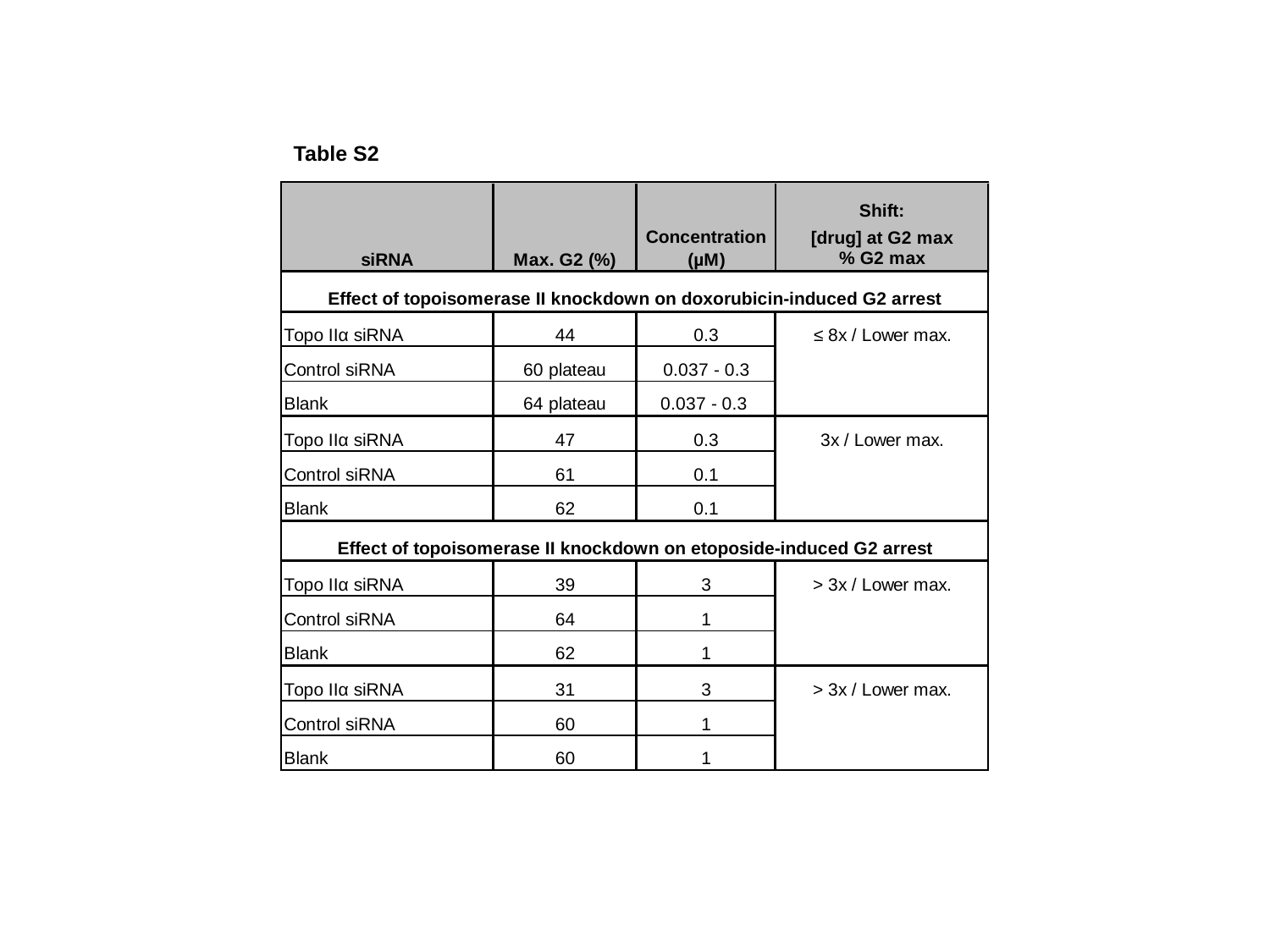

Table S2

Supplement: Table S2 — Summary of additional two experiments investigating the effect of topoisomerase IIα knockdown on doxorubicin and etoposide-induced G2 arrests. Indicated for each treatment group are the drug concentrations at which maximal G2 arrest was observed, and the percentage of cells in G2 at maximal arrest for each treatment group. Each experiment was performed independently. (0.12 MB PPT) [file pone.0010186.s008.ppt]

## Slide 1
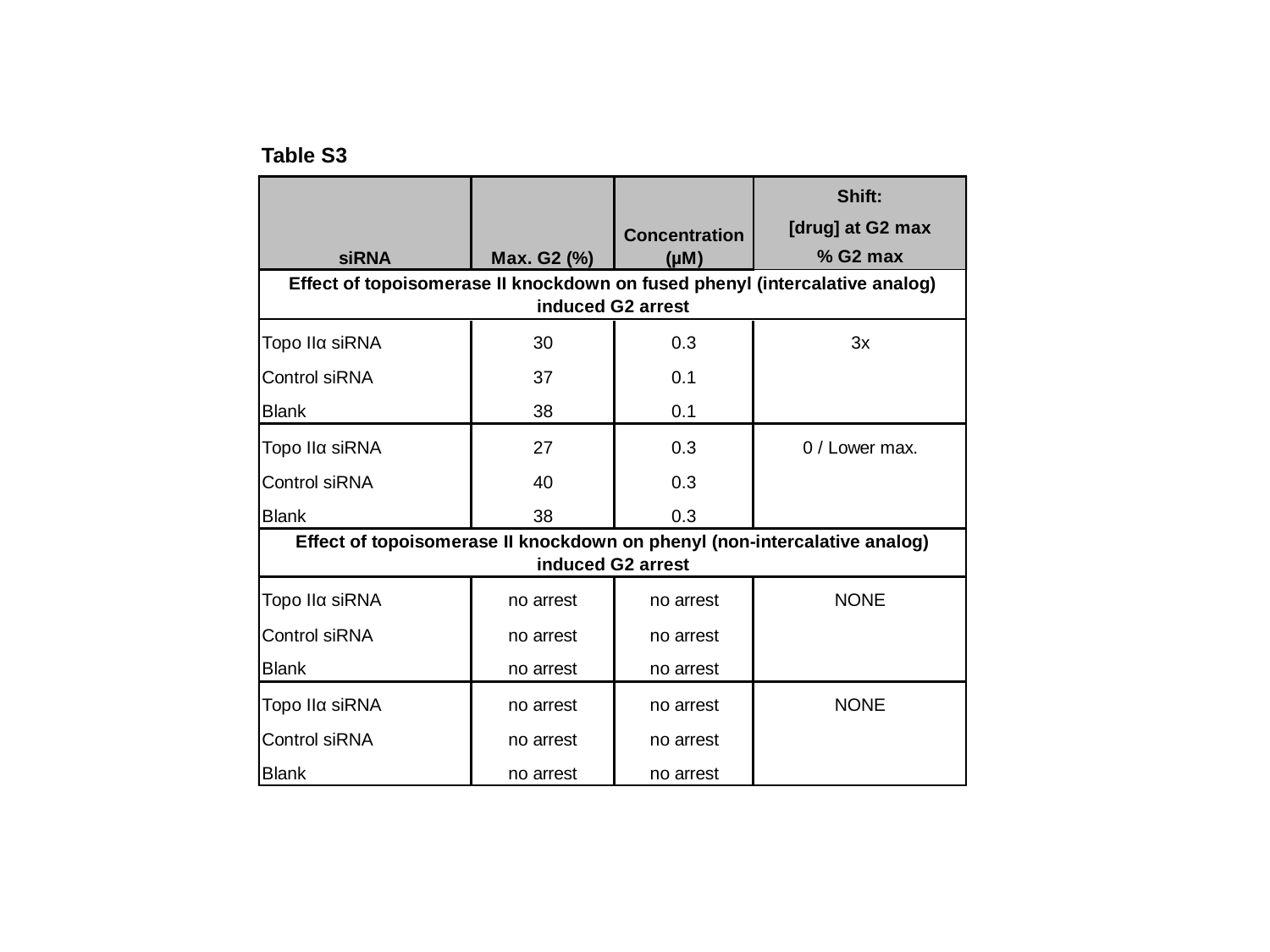

Table S3

Supplement: Table S3 — Summary of additional two experiments investigating the effect of topoisomerase IIα knockdown on the planar analog and phenyl analog-induced G2 arrests. Indicated for each treatment group are the drug concentrations at which maximal G2 arrest was observed, and the percentage of cells in G2 at maximal arrest for each treatment group. Each experiment was performed independently. (0.12 MB PPT) [file pone.0010186.s009.ppt]
