# Supplementary material for: Voreloxin Is an Anticancer Quinolone Derivative that Intercalates DNA and Poisons Topoisomerase II
Source: PLoS One. 2010 Apr 15;5(4):e10186. doi: 10.1371/journal.pone.0010186 (PMC2855444; doi:10.1371/journal.pone.0010186)
Supplement: Methods S1 — (0.04 MB DOC) [file pone.0010186.s010.doc]

**SI Methods**

**Pulsed-Field Gel Electrophoresis (PFGE).** CCRF-CEM human leukemic cells were treated with different concentrations of voreloxin for 6 h. Untreated, vehicle-treated (0.1% final concentration), and doxorubicin-treated (0.1 μM) samples were included as controls. Cells were then washed once with PBS, suspended in PBS, and mixed 1:1 with 2% low-melting agarose solution to a final concentration of 1.25 X 10^6^ cells per 0.1 mL of agarose block. The blocks were solidified at 4°C for 30 min, and the agarose-embedded cells were then lysed in 100 mmol/L of EDTA (pH 8.0), 1% sodium lauryl sarcosine, and 1 mg/mL of proteinase K for 48 h at 50°C. The samples were then washed thrice with 10 mmol/L of Tris (pH 7.5), 1 mmol/L EDTA, and 1 mg/mL of RNase A for 10 min at 37°C. The DNA agarose plugs were inserted into 1% Pulsed Field Certified Agarose gels and DNA was separated by electrophoresis using CHEF Mapper System (Bio-Rad Laboratories). The gel was run using parameters derived by algorithm for separations from 5-150 kb, and electrophoresis was done at 14°C for 15 h with the following parameters: initial switch time was 0.23 s, final switch time was 12.9 s, 120 degree angle, 6 V/cm linear gradient. After electrophoresis, DNA species were visualized by ethidium bromide staining under UV transillumination.

**Formation of Topoisomerase II-DNA Cleavage Complexes in Cultured Human Cells.** Human CEM leukemia cells were cultured under 5% CO_2_ at 37 °C in RPMI 1640 medium (Cellgro by Mediatech, Inc.), containing 10% heat-inactivated bovine calf serum (Hyclone) and 2 mM glutamine (Cellgro by Mediatech, Inc.). The in vivo complex of enzyme (ICE) bioassay was modified as noted on the TopoGen, Inc. web site. Exponentially growing cultures were treated for 4 h with 0.1 – 20 µM voreloxin, 1 µM doxorubicin or 1 µM and 10 µM etoposide as positive controls. Cells were harvested by centrifugation and lysed by the immediate addition of 3 mL of 1% sarkosyl. Following gentle homogenization in a dounce homogenizer, lysates were layered onto a 2 mL cushion of CsCl (1.5 g/mL) and centrifuged at 45000 rpm for 17 h at 20°C. DNA pellets were isolated, resuspended in 5 mM Tris-HCl (pH 8.0) and 0.5 mM EDTA, normalized for DNA content, and blotted onto nitrocellulose membranes using a Schleicher and Schuell slot blot apparatus. Covalent complexes formed between human topoisomerase IIα or IIβ and DNA were detected using a polyclonal antibody directed against either human topoisomerase IIα or IIβ (Abcam), at a 1:1000 dilution.

**Site-specific DNA Cleavage Mediated by Topoisomerase II.** A linear 4330 bp fragment (*Hin*dIII/*Eco*RI) of pBR322 plasmid DNA singly labeled with ^32^P on the 5'-terminus of the *Hin*dIII site was used as cleavage substrate. Terminal 5'-phosphates were removed by treatment with calf intestinal alkaline phosphatase and replaced with [^32^P]phosphate using T4 polynucleotide kinase and [*γ*-^32^P]ATP. DNA was treated with *Eco*RI, and the 4330 bp singly end-labeled fragment was purified from the small *Eco*RI-*Hin*dIII fragment by passage through a CHROMA SPIN+TE-100 column (Clontech). Reaction mixtures contained 1.4 pM labeled pBR322 DNA substrate and 60 nM human topoisomerase II in 50 µL of 10 mM Tris-HCl (pH 7.9), 5 mM MgCl_2_, 100 mM KCl, 0.1 mM EDTA, 0.4 mM ATP, and 2.5% (v/v) glycerol. Assays were performed using 0–10 µM voreloxin and were incubated for 0.5 min at 37°C. Cleavage intermediates were trapped by adding 5 µL of 5% SDS followed by 3.75 µL of 250 mM EDTA (pH 8.0). Topoisomerase II was digested with proteinase K (5 µL of a 0.8 mg/mL solution) for 30 min at 45 °C. DNA products were precipitated twice in 100% ethanol, washed in 70% ethanol, dried, and resuspended in 6 µL of 40% formamide, 10 mM NaOH, 0.02% xylene cyanol FF, and 0.02% bromophenol blue. Samples were subjected to electrophoresis in a denaturing 6% polyacrylamide sequencing gel; 100 mM Tris-borate pH 8.3, and 2 mM EDTA. The gel was dried and the DNA cleavage products analyzed on a Bio-Rad Molecular Imager FX.

**DNA Intercalation.** Iintercalative agents induce constrained negative supercoils and compensatory unconstrained positive superhelical twists in covalently closed circular DNA. As the concentration of an intercalative compound increases, a plasmid that is negatively supercoiled or relaxed (i.e., contains no superhelical twists) appears to become positively supercoiled. Treatment of an intercalated plasmid with topoisomerase I removes the unconstrained positive DNA superhelical twists. Subsequent extraction of the compound allows the local drug-induced unwinding to redistribute in a global manner and manifest as a net negative supercoiling of the plasmid. Thus, in the presence of an intercalative agent, topoisomerase treatment converts relaxed plasmids to negatively supercoiled molecules.

**Colony formation assay.** A549 cells were seeded at 500 cells/well in a 6-well dish and incubated overnight. The cells were then treated with a dose-titration of either voreloxin, the fixed-ring analog, or the phenyl analog, all ranging from 37 nM to 3 µM. Following 16 h of treatment, the compound-containing media was removed and the wells washed twice with normal growth media. Normal growth media was then added to the wells and the cells were allowed to continue growth. After 5 days, the media was removed and the cells were fixed with 4% formaldehyde in PBS. The resulting colonies were counted using a Cellomics ArrayScan high content screening device. In order to identify cells on the ArrayScan, nuclei were stained with Hoechst 33342, and colonies counted using the Cellomics Morphology Explorer protocol. Cell survival was plotted as a percentage of colony formation compared to a vehicle control.

**Cell Cycle Analysis.** A549 cells were treated with 0.001 - 3 μM drug diluted in RPMI 1640 growth media containing 10% fetal calf serum for 16 h. Adherent cells were detached with 0.1% trypsin-EDTA solution, combined with floating cells, and pelleted by centrifugation for 5 min at 300 x *g*. Cell pellets were washed once with PBS and fixed in 80% methanol. Cells were centrifuged for 5 min at 300 x *g* and the methanol was removed. The cell pellet was washed once with PBS containing 1% BSA (Sigma) and incubated in PBS containing 1% BSA, 10 µg/mL propidium iodide, 100 µg/mL RNase A, and 0.1% Triton X-100. Cells were analyzed by FACS (BD) for total DNA content based on propidium iodide fluorescence. Ten thousand cells were analyzed per treatment.

**siRNA knockdown.** A549 cells were transfected with 75 nM topoisomerase IIα-targeting siRNA (Dharmacon) combined with Lipofectamine 2000 (Invitrogen) in RPMI 1640 growth media containing 10% fetal calf serum, as recommended by the manufacturers. Additional control samples included cells transfected with nontargeting siRNA (Dharmacon) and cells treated with Lipofectamine 2000 alone. After 24 h, cells were harvested with 0.1% trypsin-EDTA and seeded in 12-well dishes at 50,000 cells/well in normal growth media. Following an additional 24 h of growth (48 h following the initial exposure to siRNA), cells were treated with a dose-titration of voreloxin, doxorubicin, etoposide,or voreloxin analog.

**Western blot.**

Cell pellets were washed once in 4°C PBS and lysed in M-PER buffer (Pierce) containing phosphatase (Sigma-Aldrich) and protease inhibitors (Roche Applied Science). Lysates were normalized for total protein content and 10 μg total protein electropheresed on a 4-20% Bis-Tris gel (Invitrogen) followed by transfer to a PVDF membrane (Invitrogen). The membrane was blocked for 1 hour in 5% non-fat dry milk (NFDM) in TBS with 0.1% Tween-20 (TBST) before overnight incubation in 5% NFDM at 4°C with primary antibodies (anti-topoisomerase IIα, Abcam #12318 diluted 1:1000 or anti- beta actin Sigma #A2228, diluted 1:40000) . Following three TBST washes, blots were probed for 1 hour with HRP-conjugated secondary antibody (Zymed #62-6520, 62-6120) diluted 1:10000 in 5% NFDM-TBST, washed as previously and developed using ECL developing solution (Amersham), according to the manufacturer’s protocol. The blots were then exposed to x-ray film (Kodak) and images acquired using a flatbed scanner (Epson, Long Beach, CA, USA).
